# Serum Metabolic Profiling Identifies a Biomarker Panel for Improvement of Prostate Cancer Diagnosis

**DOI:** 10.3389/fonc.2021.666320

**Published:** 2021-05-07

**Authors:** Huan Xu, Junyi Chen, Jingyi He, Jin Ji, Zhi Cao, Xi Chen, Yalong Xu, Xing He, Guowang Xu, Lina Zhou, Xuedong Wei, Jianquan Hou, Zhong Wang, Bo Yang, Fubo Wang

**Affiliations:** ^1^ Department of Urology, Shanghai Changhai Hospital, Second Military Medical University, Shanghai, China; ^2^ Department of Urology, Shanghai Ninth People’s Hospital, Shanghai, China; ^3^ Department of Urology, The Second Affiliated Hospital of Fujian Medical University, Quanzhou, China; ^4^ Department of Urology, The First Affiliated Hospital of Soochow University, Jiangsu, China; ^5^ Dalian Institute of Chemical Physics, Chinese Academy of Sciences, Dalian, China

**Keywords:** prostate cancer, metabolic profiling, early diagnosis, biomarker, metabolic panel

## Abstract

**Objectives:**

To identify and validate a biomarker panel by serum metabolic profiling for improvement of PCa diagnosis.

**Materials and Methods:**

Totally, 134 individuals were included in this study. Among them, 39 PCa patients and 45 control patients (negative prostate biopsy) were involved in the discovery phase and 50 healthy controls were enrolled for validation phase of metabolomics study. LC-MS Analysis was used for the identification of the serum metabolites of patients.

**Results:**

Logistics regression analysis shows that 5 metabolites [dMePE(18:0/18:2), PC(16:0/20:2), PS(15:0/18:2), SM(d16:0/24:1], Carnitine C14:0) were significantly changed in PCa patients compared with control patients. A metabolic panel (MET) was calculated, showing a significantly higher diagnostic performance than PSA in differentiating PCa from control patients [AUC (MET *vs*. PSA): 0.823 ± 0.046 *vs*. 0.712 ± 0.057, p<0.001]. Moreover, this panel was superior to PSA in distinguishing PCa from negative prostate biopsies when PSA levels were less than 20 ng/ml [AUC (MET *vs*. PSA]: 0.836 ± 0.050 *vs*. 0.656 ± 0.067, p<0.001]. In the validation set, the MET panel yielded an AUC of 0.823 in distinguishing PCa patients from healthy controls, showing a significant improvement of PCa detection.

**Conclusions:**

The metabolite biomarker panel discovered in this study presents a good diagnostic performance for the detection of PCa.

## Introduction

Prostate cancer (PCa) is the second most common malignant tumor in the male worldwide (1). Although the prognosis for PCa at early stage is well performed, the prognosis for patients diagnosed at an advanced stage is poor (2). Now, the early detection mainly depends on the level of prostate specific antigen (PSA) followed by prostate biopsy which is the gold standard for the PCa diagnosis. However, only 25% of men who take prostate biopsy due to an elevated PSA are proved to have PCa after the prostate biopsy (3). Therefore, it is still urgent to discover novel biomarkers or panels for the improvement of PCa diagnosis.

Circulation biomarkers is of great importance for the PCa diagnosis which can facilitate understanding of tumor biology and development (4). The peripheral blood based liquid biopsy is a powerful tool being used for the discovery of novel biomarkers for many diseases, including malignant tumors. More importantly, it provides evidences for the better understanding of the initiation and development of diseases. As one of the most widely used liquid biopsy, metabolic profiling plays significant role in the cancer diagnosis. As is known, metabolism reprograming is the key signature of cancer, which can be driven by oncogenes. Moreover, the metabolite has also been shown to change the gene expression epigenetically. The metabolites can be produced by tumors or regulated by the whole-body condition, which has been proved to change the gene epigenetic modification (5). Otherwise, metabolic factors themselves are able to change the disease progression (6). In this way, the serum metabolite is not only the result but also the reason for cancer development. Thus, the metabolomic panel can be employed as a novel approach to gain further insight into cancer diagnosis and potential therapeutic targets. Though our group having presented the metabolic profiling of the analysis between PCa tissues and adjacent normal tissues (ANT) (7, 8), there is still no such studies to investigate the metabolic changes in circulation, to our knowledge. Moreover, the spectrometry technology has been improved a lot recently. More details of molecular structure tend to be observed by using this method and specific protocol (9–11). For example, the use of nanoflow liquid chromatography-mass spectrometry (nanoLC-MS) makes it possible to do the profiling of the glycans and the glycolipids which can be used in the clinic studies, including glycan-based biomarker discovery and therapeutics.

In this study, we used the GC-MS based metabolomics, LC-MS based metabolomics and LC-MS based lipidomic for the analysis of serum metabolites, which might provide a novel method for PCa detection and a better understanding of the metabolic reprogramming of PCa.

## Materials and Methods

### Sample Collection

This project was approved by the Clinical Research Ethics Committee of Shanghai Changhai Hospital of Second Military Medical University. All of the clinical samples were obtained from Shanghai Changhai Hospital (Shanghai, China). Written informed consents were obtained from the participants before sampling.

Totally, 39 PCa patients and 45 control patients were involved in the discovery phase and 50 healthy controls were enrolled for validation phase of metabolomics study, respectively. The samples of three groups ran at the same time and analyzed separately. Blood samples were collected from patients undergoing prostate biopsy from January 2018 to November 2018. Patients who underwent biopsy were assessed to have a PSA increase greater than 4 ng/ml or PSA that was not elevated, but the rectal examination revealed a nodule or imaging examination abnormality. On the first day of admission, each patient signed an informed consent form, the label was printed and attached to the BD serum collection tube. On the second morning of admission, 3 to 5 ml of fasting peripheral blood was collected from the patient. Samples were stored at 4°C, transported to the laboratory with ice to separate the serum, centrifuged at 1500*g* for 20 min, and the serum was collected. The serum was placed in a 1.5 ml centrifuge tube and numbered with the admission number and specimen type. All of the serum was immediately stored at −80°C until further processing to make sure the metabolites were unchanged before analysis. The hematoxylin and eosin (H&E) stained slides of specimens after biopsies were examined by two pathologists to confirm the diagnosis and Gleason Score.

## Three Platforms For The Analysis Of Serum Metabolites

### Reagent

Internal standards, including lysophosphatidylcholine C19:0 (LPC 19:0), phosphatidylcholine C38:0 (PC 38:0), phosphatidylethanolamine C30:0 (PE 30:0), sphingomyelin C12:0 (SM 12:0), ceramide C17:0 (Cer 17:0), fatty acid C16:0-d3 (FA 16:0-d3), fatty acid C18:0-d3 (FA 18:0-d3) and triglyceride 45:0 (TG 45:0) were purchased from Avanti Polar Lipids (Alabaster, AL). Internal standards, including acetylcarnitine-d3 (carnitine C2:0-d3), carnitine C10:0-d3, carnitine C16:0-d3, tryptophan-d5, phenylalanine-d5, cholic acid-d4, chenodeoxycholic acid-d4) were purchased from Sigma company (US). Ammonium acetate and tert-butyl methyl ether (MTBE) were purchased from Sigma company (US). Acetonitrile, methanol and isopropanol were purchased from Merk company (German). Milli-Q water was made from a Milli-Q system (Millipore,Billerica,MA). Pyridine, methoxyamine hydrochloride, and N-Methyl-N-(trimethylsilyl) trifluoroacetamide (MSTFA) for GC-MS derivatization were obtained from Sigma-Aldrich (St. Louis, MO, USA).

### Sample Preparation

For GC-MS based metabolomics. The serum samples were thawed at room temperature and vortex for 5 sec. Then 50 μl serum sample was drawn into to 1.5 ml Eppendorf tubes, and 200 μl methanol containing internal standard (10 μg/ml tridecanoic acid) was added subsequently for metabolite extraction and protein precipitation. After full vortex for another 5 min at room temperature, the supernatant was removed and lyophilized in new 1.5 ml Eppendorf tubes. Each aliquot was reconstituted in 50 μl methoxyamine pyridine solution (20 mg/ml). After full vortex, the tubes with the solutions were incubated in water bath at 37°C for 1.5 h. Then, silylation reaction lasted for 1 h at 37°C in water bath after adding 40 μl of MSTFA. The tubes were centrifuged at 13,000*g* for 10 min at 4°C, and the supernatants were drawn for GC-MS injections.

For LC-MS based metabolomics. A 96-well filter protein precipitation plate was used for sample preparation. An aliquot of 50 μl serum was drawn into each well, and 200 μl extraction solvent was added subsequently for metabolite extraction and protein precipitation. This extraction solvent was methanol containing internal standards (0.5 µg/ml fatty acid (FFA) C16:0-d3, 0.5 µg/ml FFA C18:0-d3, 0.16 µg/ml carnitine C2:0-d3, 0.1 µg/ml carnitine C10:0-d3, 0.075 µg/ml carnitine C16:0-d3, 0.75 µg/ml LPC 19:0, 4.25 µg/ml tryptophan-d5, 3.61 µg/ml phenylalanine-d5, 0.37 µg/ml cholic acid-d4, 0.3 µg/ml chenodeoxycholic acid-d4). Then the plate was covered with aluminum foil and vortexed for 10 min, then it was centrifuged for collecting supernatant. The extracts were lyophilized and stored at −80°C before LC-MS analysis.

For LC-MS based lipidomics. An aliquot of 40 μl of serum for each sample was drawn into Eppendorf tube. And 300 μl of methanol containing LPC 19:0 (0.80 μg/ml), PC 38:0 (1.00 μg/ml), PE 30:0 (0.75 μg/ml), SM 12:0 (0.65 μg/ml), Cer 17:0 (0.80 μg/ml), FFA 16:0-d3 (0.50 μg/ml), FFA18:0-d3 (0.20 μg/ml), TG 45:0 (0.60 μg/ml) was added, followed by vortex mixing for 30 sec and protein denaturation. Then, 1 ml of MTBE and 250 μl of water were added into the mixture followed by vortex mixing for 30 sec to extract lipid compounds. After centrifugation at 14000 rpm at 10°C for 10 min, 400 μl of organic supernatant was collected and lyophilized, then stored at −80°C.

### Data Acquisition

For GC-MS based metabolomics. One microliter of the above derivatized products were injected into a gas chromatograph coupled to a plus quadrupole mass spectrometer detector (GCMS 5977A; Agilent, US). The split ratio of 1:10 was employed. A DB-5 MS capillary column (length 30 m × ID 0.25 mm, film thickness 0.25 μm) (J&W Scientific, Folsom, CA, USA) was employed for metabolite separation. The flow rate of the helium gas was 1 ml/min; the oven temperature began at 80°C and maintained for 1 min, which was linearly increased to 210°C at 30°C/min, and then linearly increased to 320°C at 20 °C/min, and holding for 4 min at 320°C. The temperature of the ion source was 230°C. The data was acquired in full scan mode with the scan range set at 33 to 600 Dalton.

For LC-MS based metabolomics. The dried powder was reconstituted in 80 μl acetonitrile/water (1:4) and centrifuged at 13 000 g for 10 min at 4°C. 5 μl of the supernatant was injected into a Vanquish UPLC- Q Exactive (Thermo Fisher Scientific, Rockford, IL, USA) system. For positive ionization mode, a 50 mm × 2.1 mm, 1.7 μm Waters BEH C8 (Waters, Milford, MA) column was used for separation; Mobile phase A was 0.1% formic acid in water and mobile phase B was 0.1% formic acid in acetonitrile. The elution gradient program started from 5% B and kept for 0.5 min, then linearly increased to 40% B within 1.5 min, continued to 100% B in 6 min and maintained at 100% B for 2 min, then back to 5% B within 0.1 min. A post- equilibration was kept for 2.5 min. For negative ionization mode A, a 50 mm × 2.1 mm, 1.8 μm ACQUITY UPLC HSS T3 (Waters, Milford, MA) column was used for separation. Mobile phase A was 6.5 mM ammonium bicarbonate in water and mobile phase B was 6.5 mM ammonium bicarbonate in 95% methanol/water. The elution gradient program started from 2% B and kept for 0.5 min, then linearly increased to 40% B within 2 min, and further increased to 100% B within another 6 min and maintained at 100% B for 2 min, then back to 2%B in 0.1 min. A post-equilibration was maintained for 2 min. In both positive and negative ionization mode, the oven temperature was 60 °C and the flow rate was 0.4 ml/min. For MS detection, resolution was set at 120 K and full scan mode was employed with m/z scan range 80 to 1200. The spray voltage was 3.5 kV for positive ionization mode and 3.0 kV for negative ionization mode, respectively. The capillary temperature was 300 °C and auxiliary gas heater temperature was set at 350°C. The flow rates of sheath gas and auxiliary gas were set at 45 and 10 in arbitrary units.

For LC-MS lipidomics. The dried sample was reconstituted in 30 μl chloroform/methonal (v/v, 2:1) and 60 μl acetonitrile/isopropanol/MilliQ water (v/v/v, 65: 30: 5). An aliquot of 5 μl reconstituted solution was injected into a Waters UPLC system (ThermoFisher, US) coupled to a Q Exactive™ HF (ThermoFisher, US). A ACQUITY UPLC C8 column (100 mm × 2.1 mm × 1.7 μm) was used for separation. Mobile phase A was 60% acetonitrile in water containing 10 mM ammonium acetate. Mobile phase B was 10% acetonitrile in isopropanol containing 10 mM ammonium acetate. Elution gradient started with 32% B and kept for 1.5 min. Then it was linearly up to 85% within next 14 min, then to 97% B within 0.1 min and kept for 2.4 min. The gradient was back to 32% B within next 0.1 min and kept for 1.9 min. Flow rate was set at 0.26 ml/min. Column temperature was 55 °C. For MS detection, resolution was set up 120 K and full scan mode was employed. The scan range was 200 to 1,100 Da for positive ionization mode, 120 – 1600 Da for negative ionization mode. The spray voltage was 3.5 kV for positive ionization mode and 3.0 kV for negative ionization mode, respectively. The capillary temperature was 300 °C and auxiliary gas heater temperature was set at 350 °C. The flow rates of sheath gas and auxiliary gas were set at 45 and 10 in arbitrary units.

### Data Preprocessing

For GC-MS metabolomics data preprocessing, to increase the detection of the low-abundance metabolites, two-fold QC samples was used for metabolite identification *via* library searching (NIST). Both comparison of mass spectrum similarity and retention index distance of reference standards in our in-house library were employed for metabolite identification. Then, the identified table was used for the next peak integration by MassHunter Workstation software (Agilent, US).

For LC-MS metabolomics data preprocessing, after automated peak detection, alignment, and integration, peaks were identified according to our in-house database containing more than 2,000 metabolite standards.

For lipidomics data preprocessing, lipid identification was performed followed by peak integration for individual lipid molecule. Lipid Search™ 4.1 (ThermoFisher, US) was employed to obtain general information of lipid candidates, including m/z, retention time, (sub)class, feature fragments and mass accuracy (in ppm). Then, manual check d for detailed structural information and known peak extraction were performed. The details in annotation can be referred to our previous publication (12).

Mass spectrometry responses of metabolites or lipids were normalized to internal standards. For LC-MS metabolomics and lipidomics analyses, internal standards were chosen firstly according to their corresponding classes. If no, then internal standards were chosen according to similarity in structure or retention time.

### Statistical Analysis

The correlations between pre-surgical characteristics and perioperative outcomes were assessed by using Pearson and Spearman correlation analysis. Logistics analysis was performed to estimate the different metabolite level and the PCa diagnosis. Moreover, the receiver operating characteristic (ROC) curves were plotted to assess the predictive ability of the panel we got in the discovery study. In the validation set, One-way ANOVA analysis, t-test and ROC were used. All analysis was performed by using SPSS, version 19.0 (IBM Corp., Armonk, NY, USA) and all tests of statistical significance were based on two-side probability.

## Results

### Study Design

**Figure 1:**
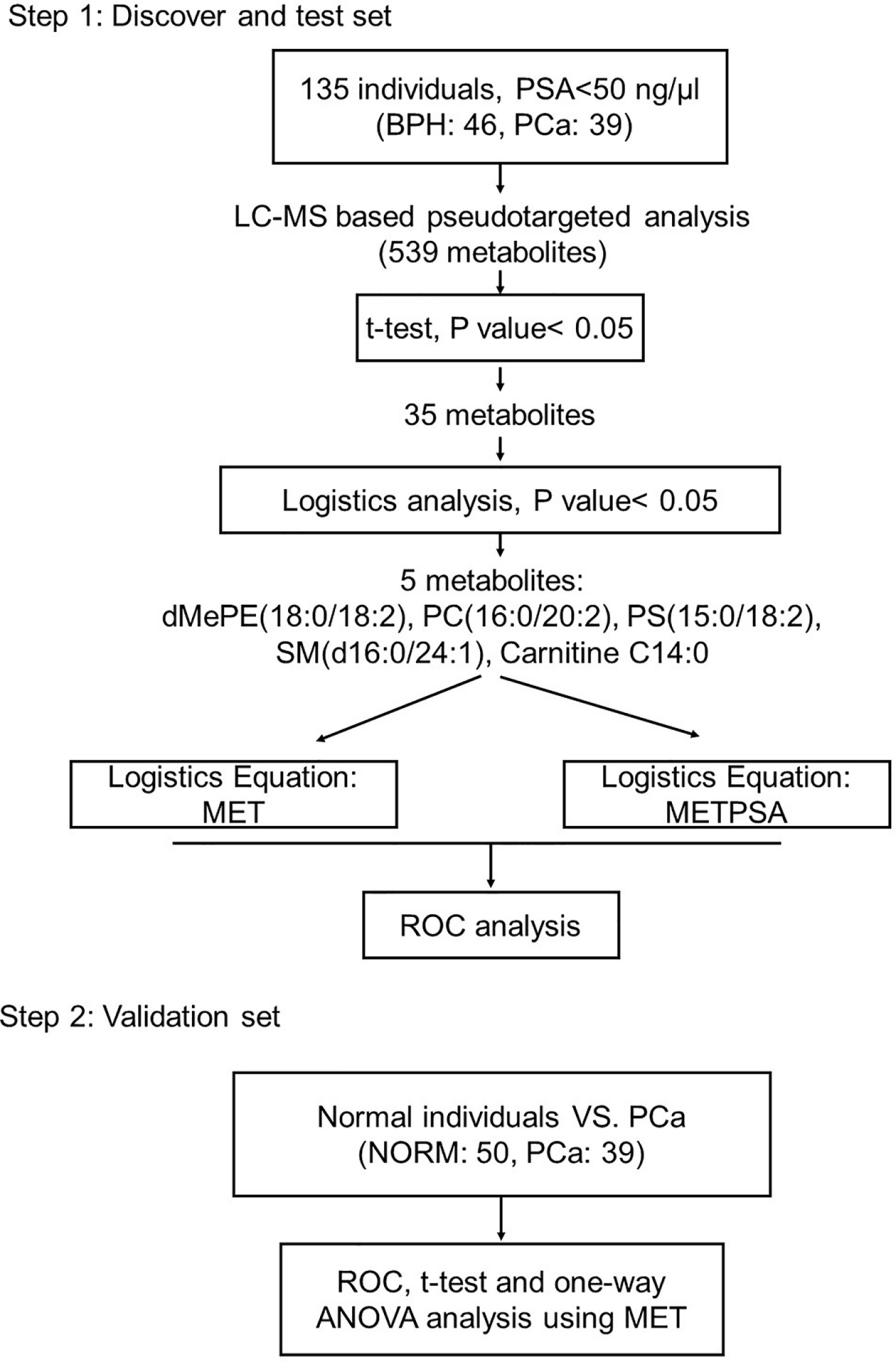
Design of the study.

There are two steps in this study. The first step is discovery and test set and the second is validation set. In the discovery set, 85 individuals, including 46 BPH and 39 PCa patients, were recruited for the discovery of the significant biomarkers. Totally, there 539 metabolites involved in this analysis. 35 metabolites (shown in **Supplemental Table**) were selected to be significantly changed with t-test and the P value< 0.05. All of the 35 metabolites were analyzed with Logistics regression analysis and 5 metabolites (dMePE [18:0/18:2], PC [16:0/20:2], PS [15:0/18:2], SM [d16:0/24:1], Carnitine [C14:0]) were significantly changed accordingly. A metabolic panel (MET) was calculated using the metabolites above. In the validation set, 50 normal individuals were enrolled and MET was confirmed between normal individuals and PCa patients enrolled in the first step. The workflow is shown in **Figure 1**.

### Individual Characteristics

There was no significant difference in the distribution of age between the PCa and BPH individuals (68.8 ± 7.7 *vs*. 65.6 ± 7.3, p=0.083). The normal individuals were enrolled from Physical Examination Center with the age 57.2 ± 10.3. Total PSA level (tPSA) in PCa patients is 15.6 (8.6–21.3) ng/ml and 9.7 (5.7–11.8) ng/ml in BPH patients with the p value less than 0.001. In the 39 PCa patients, 11 patients are with the Gleason’s Score (GS) =6, 12 individuals with GS =7, 15 patients with the GS level ≥8 and 1 patient’s GS data was missing.

### Establishing the Metabolic Panel for PCa

**Figure 2:**
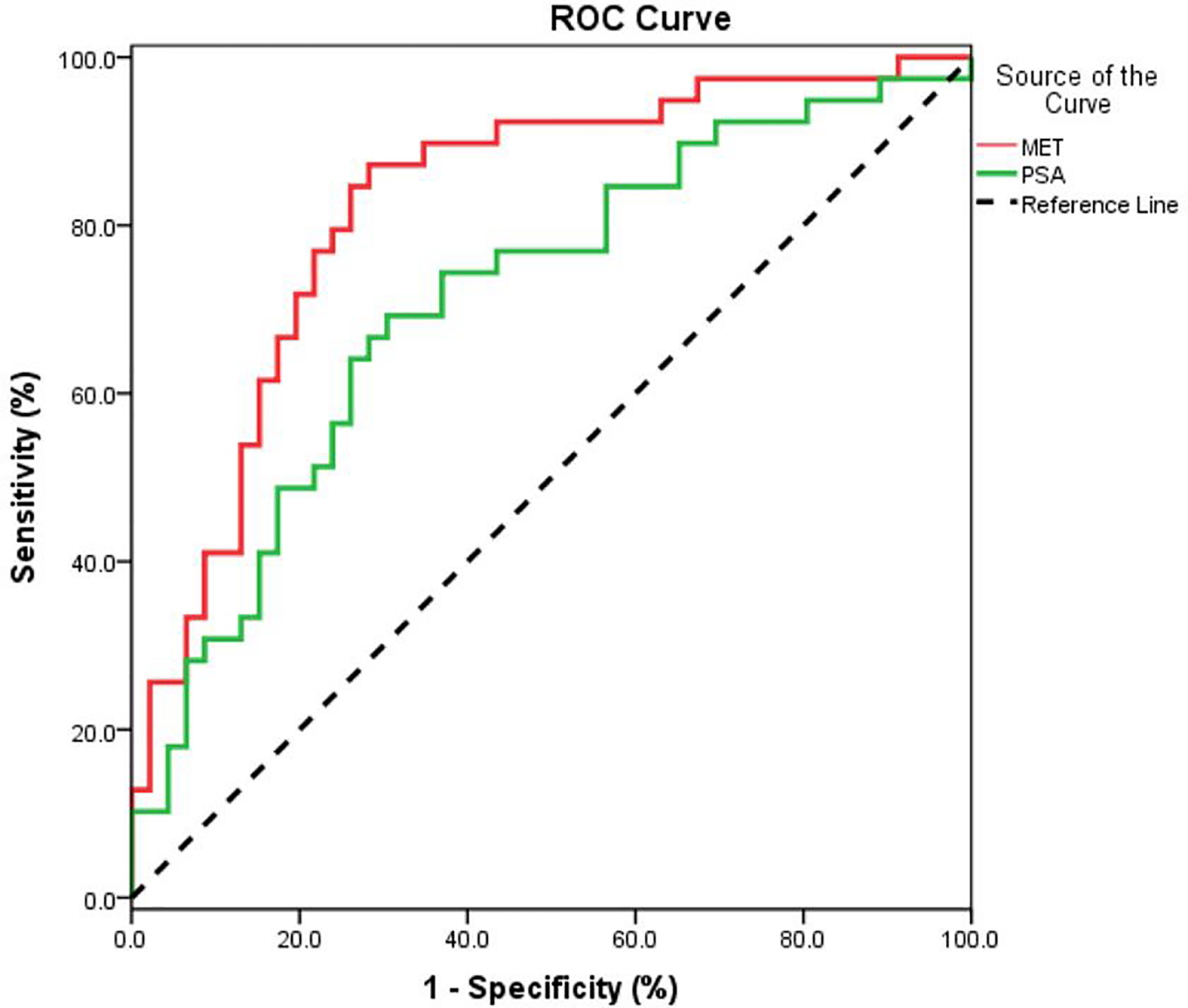
The ROC curve for PCa detection using PSA or MET methods. A metabolic panel (MET) consists of dMePE (18:0/18:2), PC (16:0/20:2), PS (15:0/18:2), SM(d16:0/24:1) and Carnitine C14:0).

**Figure 3:**
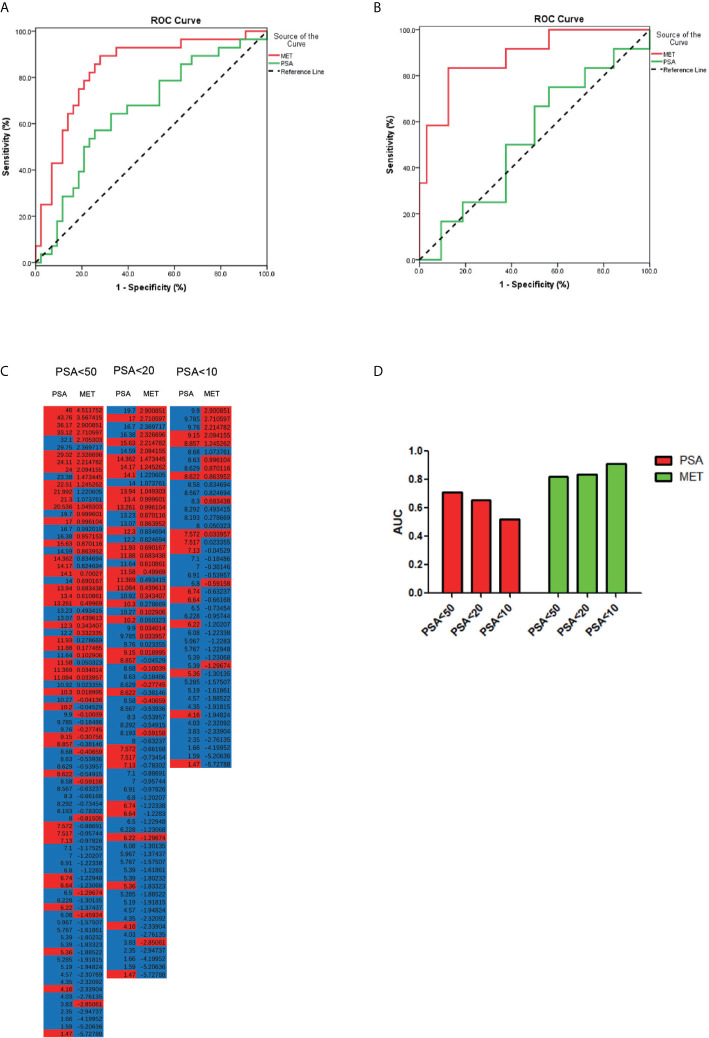
The PCa detection in different PSA levels. **(A)** ROC curve for the patients with PSA level less than 20 ng/μl. **(B)** ROC curve for patients with PSA level less than 10 ng/μl. **(C)** The accuracy of diagnosis using serum PSA or MET panel in different PSA levels. **(D)** The AUC of PCa diagnosis using PSA or MET panel in different PSA levels.

Data from quality control (QC) samples shows that the present analysis was stable and repeatable. A stepwise logistic regression model to estimate the risk of being diagnosed with PCa was applied on this data set. All of the metabolites turned out to be significant predictors. Logit (p=PCa) =0.211*dMePE (18:0/18:2)-0.587* PC (16:0/20:2)-0.173* PS (15:0/18:2)-1.59* SM(d16:0/24:1) +334.9*Carnitine (C14:0) +12.299 was used for the ROC curve. The diagnostic performance for the established metabolism panel was evaluated by using ROC analysis. The MET panel showed a higher diagnostic performance than the PSA in differentiating PCa from control patients [AUC (MET vs. PSA): 0.823 ± 0.046 *vs*. 0.712 ± 0.057, p<0.001; **Figure 2**]. Moreover, this panel was superior to PSA in distinguishing PCa from controls when PSA levels were less than 20 ng/ml [AUC (MET *vs*. PSA): 0.836 ± 0.050 *vs*. 0.656 ± 0.067, p<0.001; **Figure 3A**] or less than 10 ng/ml [AUC (MET *vs*. PSA): 0.909 ± 0.060 *vs*. 0.519 ± 0.101, p<0.001; **Figure 3B**]. The details of PSA levels with corresponding MET panel scores of each group were shown in **Figure 3C** and the AUC of PSA and MET panel of each group were shown in **Figure 3D**.

### Validating of the Metabolic Panel

**Figure 4:**
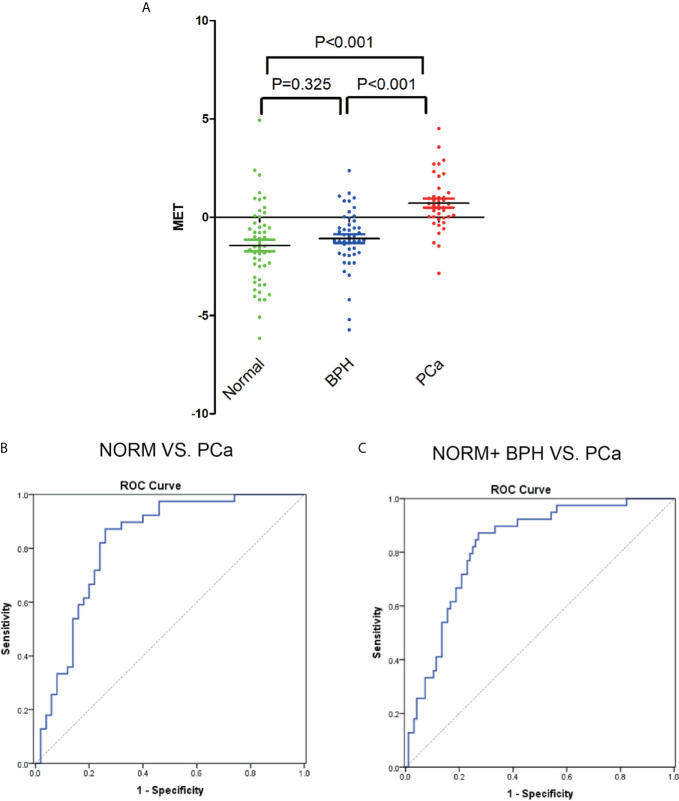
The detection of MET panel in validation phase. **(A)** ROC curve for the comparation between BPH and PCa patients. **(B)** AUC of ROC between the comparation of normal, BPH and PCa individuals. **(C)** ROC curve for the comparation between normal individuals and PCa patients.

50 samples from normal individuals collected from Physical Examination Center were included for the validation of MET panel for PCa detection. The score of MET panel in PCa is significantly higher than the score in healthy controls (p<0.001) and control patients with negative prostatic biopsies (p<0.001) while there are no significant changes between BPH and normal samples (p=0.325) according to the one-way ANOVA analysis (**Figure 4A**). Notably, this MET panel presents a favorable diagnostic performance in differentiating PCa from healthy controls (AUC, 0.823, range 0.735 to 0.911, p<0.001; **Figure 4B**). We then combined the data from control patients with normal individuals. The MET panel also yielded an AUC of 0.823 (ranging from 0.75 to 0.896) in detecting PCa from controls (**Figure 4C**).

## Discussion

Currently, the gold standard for the diagnosis of PCa is still biopsy. The traditional two main methods for the screening diagnosis of cancers are imaging and biomarker. However, the diagnostic performance of these modalities, including PSA level which is the most widely used, is unsatisfactory. In this study, we analyzed 46 BPH individuals and 39 PCa patients in the discovery and test set to get the MET pane consisting of dMePE, PC, PS, SM and Carnitine, for the diagnosis of PCa. This panel was then validated using normal individuals, which still present a high accuracy. All of these metabolites participate in the lipid metabolism, especially the phosphatidic acid metabolism, which also may indicate that phosphatidic acid in circulation may play important role in the PCa detection and development.

As is widely accepted, metabolism reprogramming is one of the most remarkable switches of cancer. In prostate cancer, lipid metabolism is rewired significantly. One of the most typical hallmarks of prostate cancer is the dependance of androgens, which has been shown to regulate the lipid metabolism through the SREBP involved pathway (13). As is accepted, the *de novo* lipogenesis is up-regulated significantly in PCa and PCa cells tend to rely more on the fatty acid oxidation process to produce energy (14–16). In recent years, the methodology for LC-MS technology is deeply developed (9–11). More details have been revealed about the target metabolite. For example, quantitative LC–tandem MS (LC-MS/MS) analysis has been used for the study of comprehensive structural glycomic characterization (11). LC-MS might be more widely used in clinic work in the future to observe the specific molecular changes in liquid biopsy.

In this study, we, for the first time, report the serum metabolite changes in PCa patients, which indicates lipid metabolism as the most significant changes among the serum metabolite profiling. Enhanced lipid metabolism is observed in PCa individuals. However, unlike many solid tumors, PCa are more dependent on lipid metabolism with increased cholesterol synthesis and steroid genesis (17, 18). In our previous study which combines metabolomics and transcriptomics profiles, we found the impaired sphingosine-1-phosphate receptor 2 signaling, downstream of sphingosine (7, 8). In addition, as the membrane synthesis in malignant cells is augmented, the phosphatidic acid in PCa tissue is significantly altered in different stages of the malignant progression. It plays critical role in the proliferation, metastasis and pathologic progression of cancer cells (19). Moreover, phosphatidic acid is also a group of metabolites participating in the cell signaling pathways. As is reported, lipid metabolism is able to regulate PI3K–mTOR signaling pathway, which involves in the oncogenic signaling pathways as well as androgen deprivation treatment (ADT) resistance (20).

The metabolites related to phosphatidic pathway are significantly altered in the serum of PCa patients according to our research. Metabolites in circulation might be affected by many different ways, including the solid tumor treatment, hormone generation and so on. In prostate cancer, phosphatidic acid may be uptake by solid tumor, which contributes to the down regulation of the serum level. Moreover, prostate cancer, especially in the early stage, is androgen-dependent, suggesting that androgen level might also affect the metabolic concentration. As is reported, androgens influence both synthesis and uptake of fatty acids in prostate cells. By activating SREBP1 which is one of the most important master of lipid metabolism, androgen gets involved in the lipid metabolism and stimulate the transcription of enzymes required for lipid metabolism (17, 21). During the development, different oncogenes play various roles in the regulation of lipid metabolism. P53, as one of the most important oncogenes, is reported to orchestrate the regulation of metabolism, especially glycolysis and lipogenesis (22). It is also observed an increased glucose uptake in c-myc cMyc/Adrb2ecKO-isolated endothelial cells, which might contribute to the lipogensis and lipid storgage (23). As a feedback, c-myc–mediated tumor genesis can be remarkably attenuated by the inhibition of CPT1 (24), which gives evidence to that oncogenes can also be influenced by the serum lipid metabolism. All of the above indicates that the tissue and serum level of various metabolites can present the cancer stage and development.

Currently, the screening of early-stage PCa remains a challenge. PSA is still the most widely used marker for PCa screening. However, men with a PSA level between 4 and 10 ng/μl have about 20% of having prostate cancer. With the PSA level being from 10 to 20 ng/ml 32.7% of those are PCa patients (25, 26). Thus, this might lead to a large group of unnecessary biopsies, for whom the PSA level over 4 ng/ml is recognized as the criteria for prostate biopsy. In the present study, we presented that the MET panel effectively discriminated patients with PCa from the control patients with negative prostatic biopsies, presenting a higher AUC than that of PSA (0.823 ± 0.046 *vs*. 0.712 ± 0.057, p=0.001). Remarkably, among the individuals with PSA level less than 20 ng/μl, this metabolite panel shows the AUC with 0.836 ± 0.050 which is significantly higher than the AUC of PSA (0.656 ± 0.067). Therefore, our results indicated that the MET panel could be served as a novel method for PCa diagnosis.

**Figure 5:**
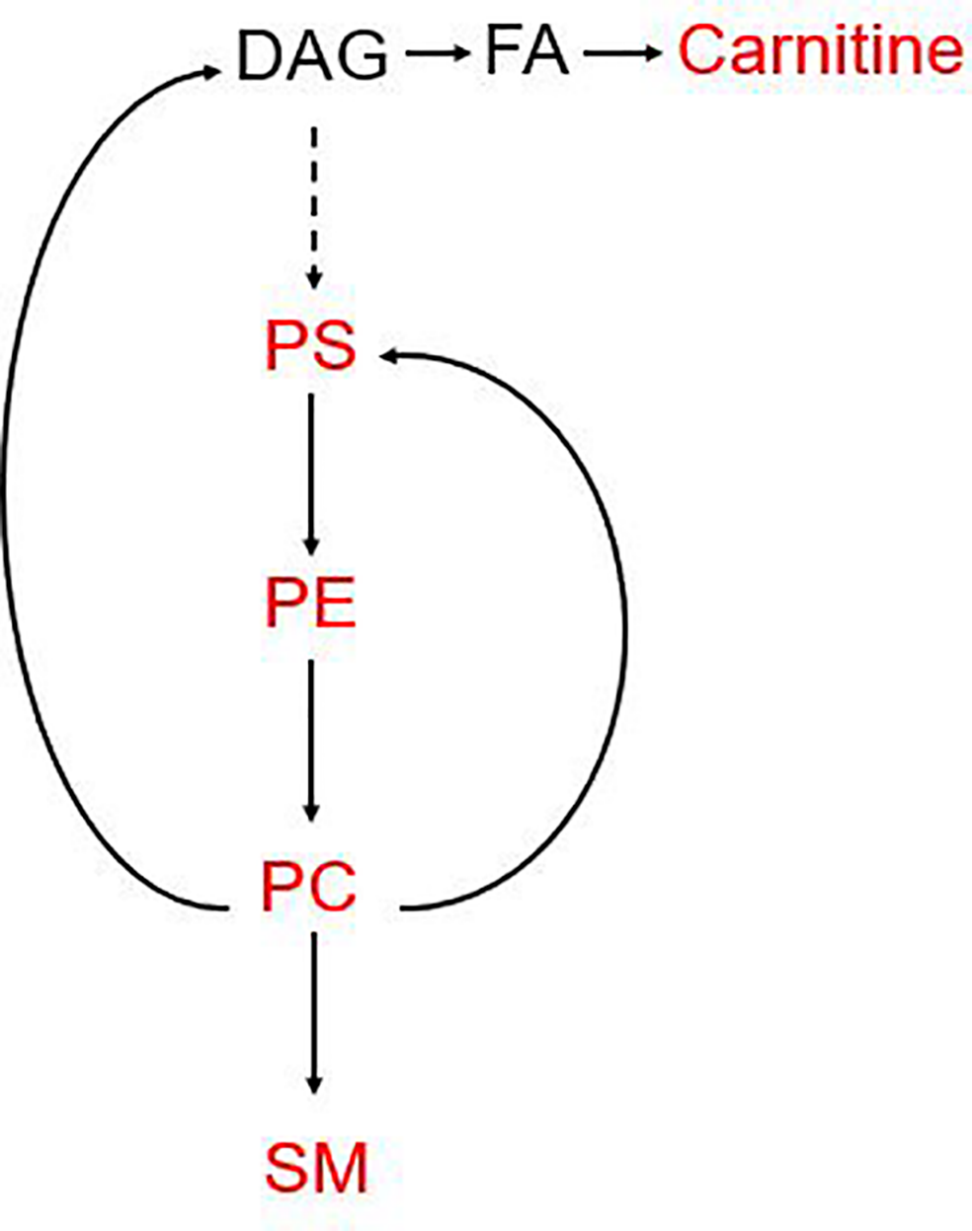
Pathways involved in the MET panel. The metabolites involved in the panel are mainly focused on the phosphorylation pathway. PC, Phosphatidylcholine; PE, phosphatidylethanolamine; SM, sphingomyelin; PS, phosphatidylserine; DAG, diacylglycerol.

Intriguingly, as is shown in **Figure 5**, all of the metabolites included in the MET panel, are gathered in the phospholipid metabolism pathway. Phosphatidylcholine (PC) and phosphatidylethanolamine (PE) are the most abundant phospholipids in cell membranes, which has been implicated in cancer metastasis, invasion and proliferation. Recently, the importance of phospholipid metabolism in regulating lipid, lipoprotein and the energy metabolism has been illustrated in *in vivo* and *in vitro* studies (19). The concentration of PC and PE regulates the size and dynamics of lipid droplets, which may affect energy production as well as the cellular signal. PC is the central metabolite in this pathway and can be made in two ways: CDP-choline pathway and PC biosynthetic pathway (PE is converted to PC) (19, 27–29). In the synthesis pathway, PS, PE, PC and SM are involved in the MET panel. The detailed mechanism for the reason why serum phospholipid metabolism related metabolites is still needed more research.

This study provides a new insight into the diagnosis of PCa. However, there still are some limitations. Firstly, this study is not a multicenter study. All the BPH and PCa individuals were collected from Changhai Hospital and the samples from normal males are enrolled from the Physical examination center. More studies should be proceeded for further validation. Secondly, PSA data for normal group were not involved in this study. Thirdly, the mechanism for the change of phosphatidic acid should stilled be further studied. Fourthly, this panel could be utilized in the diagnosis of PCa while there are no significant changes between Gleason’s Score and this panel.

## Conclusions

In conclusion, we identified and validated a novel serum metabolite panel for the diagnosis of prostate cancer, which presents good diagnostic performance for PCa detection. Meanwhile, it also provides a new perspective for a better understanding of the initiation and development of prostate cancer.

## Data Availability Statement

The raw data supporting the conclusions of this article will be made available by the authors, without undue reservation.

## Ethics Statement

The studies involving human participants were reviewed and approved by the Clinical Research Ethics Committee of Shanghai Changhai Hospital of Second Military Medical University. The patients/participants provided their written informed consent to participate in this study.

## Author Contributions

FW led the study. HX and FW contributed to the conception of the study. HX, JC, LZ, and GX performed the experiment. JJ, ZC, XC, YX, and JYH contributed significantly to analysis and manuscript preparation. HX and FW performed the data analyses and wrote the manuscript. XW, ZW, and BY helped perform the analysis with constructive discussions. JQH and XH critically reviewed the manuscript. All authors contributed to the article and approved the submitted version.

## Funding

National Natural Science Foundation of China (NSFC) (81902616 to FW), Science and Technology Support Project in the field of biomedicine of Shanghai Science and Technology Action Plan (19441909200, FW), Clinical Research Project of Shanghai Municipal Commission of Health and Family Planning (20184Y0130, FW), Precision Medicine Program of Second Military Medical University (2017JZ35, FW), Youth Startup Program of Second Military Medical University (2016QN12, FW), and Jiangsu Provincial Medical Youth Talent (QNRC2016739, XW).

## Conflict of Interest

The authors declare that the research was conducted in the absence of any commercial or financial relationships that could be construed as a potential conflict of interest.

## Supplementary Material

**Supplementary Figure 1** ROC curves for the metabolites involved in the MET panel.

## Abbreviations

ANT, adjacent normal tissues; AUC, area under curve; DAG, diacylglycerol; GC-MS, gas chromatography-mass spectrometry; H&E, hematoxylin and eosin; LC-MS, liquid chromatography-mass spectrometry PCa, prostate cancer; PC, phosphatidylcholine; PE, phosphatidylethanolamine; PS, phosphatidylserine; PSA, prostate specific antigen; SM, sphingomyelin.
