# Supplementary figures and images for: Serum Metabolic Profiling Identifies a Biomarker Panel for Improvement of Prostate Cancer Diagnosis
Source: Front Oncol. 2021 May 7;11:666320. doi: 10.3389/fonc.2021.666320 (PMC8138432; doi:10.3389/fonc.2021.666320)

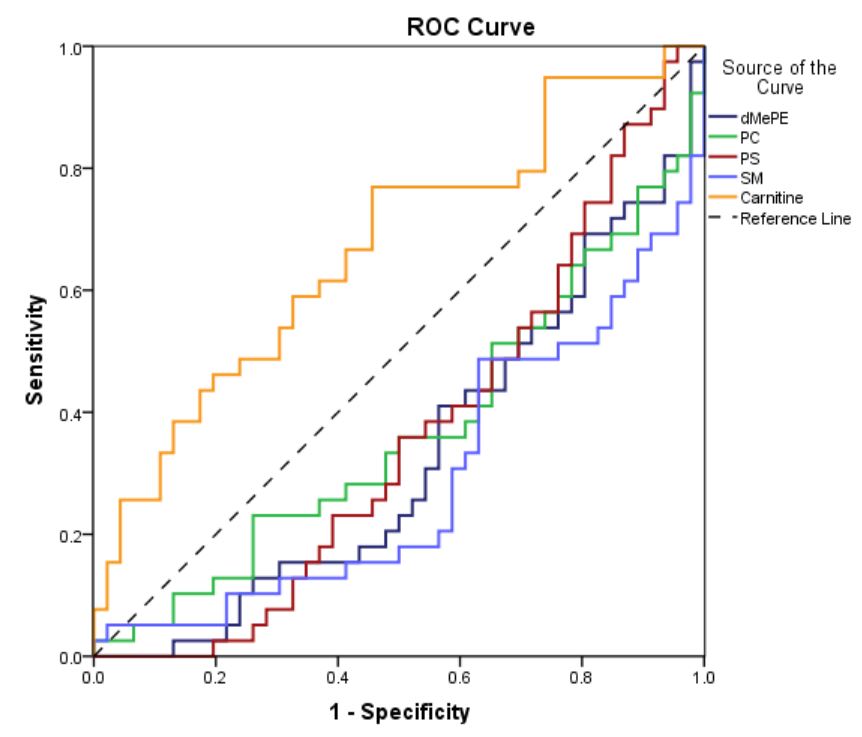

Supplement: Supplementary file 1 — Supplementary Figure 1 ROC curves for the metabolites involved in the MET panel. [file Image_1.jpeg]
